# Retina-Targeted 17β-Estradiol by the DHED Prodrug Rescues Visual Function and Actuates Neuroprotective Protein Networks After Optic Nerve Crush in a Rat Model of Surgical Menopause

**DOI:** 10.3390/ijms26051846

**Published:** 2025-02-21

**Authors:** Katalin Prokai-Tatrai, Khadiza Zaman, Ammar Kapic, Kelleigh Hogan, Gabriela Sanchez-Rodriguez, Anna E. Silverio, Vien Nguyen, Laszlo Prokai, Andrew J. Feola

**Affiliations:** 1 Department of Pharmacology and Neuroscience, University of North Texas Health Science Center, Fort Worth, TX 76107, USA; khadiza.zaman@unthsc.edu (K.Z.); ammarkapic@my.unthsc.edu (A.K.); vien.nguyen@unthsc.edu (V.N.); laszlo.prokai@unthsc.edu (L.P.); 2 Center for Visual and Neurocognitive Rehabilitation, Joseph M. Cleland Atlanta VA Medical Center, Decatur, GA 30033, USA; kelleigh.hogan@gmail.com; 3 Wallace H. Coulter Department of Biomedical Engineering, Georgia Institute of Technology and Emory University, Atlanta, GA 30332, USA; grodrquez3@gatech.edu; 4 Department of Ophthalmology, Emory Eye Center, School of Medicine, Emory University, Atlanta, GA 30322, USA; anna.silverio@emory.edu; 5 Department of Electrical and Computer Engineering, Georgia Institute of Technology, Atlanta, GA 30322, USA

**Keywords:** bioinformatics, DHED, estrogen, glaucoma, menopause, optic nerve crush, optomotor response, protein–protein interaction networks, quantitative label-free retina proteomics

## Abstract

The association between 17β-estradiol (E2) deprivation, seen in menopause, and a risk for developing glaucoma has been shown. Thus, exogenous supplementation of E2 may protect against retinal ganglion cell (RGC) degradation and vision loss. Here, we investigated the utility of topical 10β,17β-dihydroxyestra-1,4-dien-3-one (DHED), a prodrug of E2 that selectively produces the neuroprotective hormone in the retina, on visual function after optic nerve crush (ONC) and ovariectomy (OVX). We used female Brown Norway rats that underwent either Sham or OVX surgeries. After ONC, OVX animals received DHED or vehicle eye drops for 12 weeks. Visual function, via the optomotor reflex, and retinal thickness, via optical coherence tomography, were followed longitudinally. Afterward, we performed mass spectrometry-based label-free retina proteomics to survey retinal protein interaction networks in our selected animal model and to identify E2-responsive proteins after OVX on neurodegeneration. We found that ONC with OVX caused a significant decline in visual functions that were ameliorated by DHED treatments. Discovery-driven retina proteomics identified numerous proteins associated with neurodegenerative processes due to ONC that were remediated by DHED eye drops. Altogether, our three-pronged phenotypic preclinical evaluation of the topical DHED in the OVX + ONC model of glaucoma reveals the therapeutic potential of the prodrug to prevent visual deficits after glaucomatous retinal injury.

## 1. Introduction

The steroid hormone 17β-estradiol (E2) is the main human estrogen commonly known as a “female” sex hormone due to its pivotal role in reproduction and sexual maturation [1]. However, E2 also has many important functions throughout the body in both sexes [2,3,4,5]. The hormone has also been considered a neurosteroid because of its local formation in the central nervous system (CNS) [1] where it exhibits a wide range of beneficial effects [6,7]. E2 plays an important role in CNS health by protecting against neurodegeneration induced by a myriad of noxious stimuli and trauma [8,9,10]. While the neuroprotective effects of E2 have been most studied in the context of the brain [11,12,13], this hormone has also been shown to prevent neurodegeneration of other CNS structures and systems robustly [14,15,16].

Neurodegeneration is a complex, multifaceted event in terms of both initiation and progression with multiple, often interconnected, factors involved [17,18]. We have yet to fully understand these detrimental processes. However, inflammation, oxidative stress, abnormal protein aggregation, and excitotoxicity are among the clinically relevant mechanisms of action implicated in glaucoma. Therefore, pleiotropic agents such as E2 capable of acting in concert against these critical contributors of neuronal demise are needed to halt, curtail, or treat neurodegenerative processes rather than relying on polypharmacological approaches. E2’s broad-spectrum activity can thwart the onset and progression of neurodegeneration owing to its well-orchestrated genomic and rapid non-genomic actions [9,19,20,21]. There are, however, concerns regarding the direct use of the hormone for neurotherapy due to peripheral liability, such as risk for developing certain types of cancer and cardiovascular problems [13,22,23,24].

**Figure 1:**
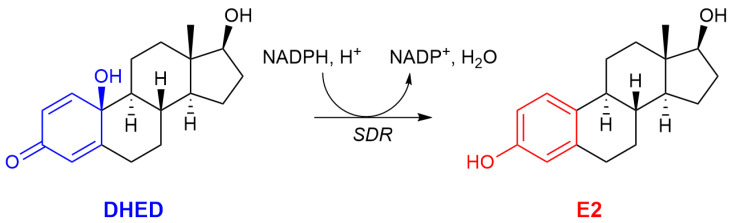
Schematic illustration of the DHED bioprecursor prodrug’s site-specific metabolism to E2 in the CNS catalyzed by a short-chain reductase (SDR) utilizing NADP(H) as a cofactor [25].

To overcome these serious limitations, we have previously reported that the unique bioprecursor prodrug DHED (10β,17β-dihydroxyestra-1,4-dien-3-one, Figure 1) metabolizes to E2 only in the CNS while remaining inert in the rest of the body [25]. Our interest lies in developing DHED for an efficacious and safe ocular neurotherapy. We focus on the retina, which is part of the CNS, with special emphasis on glaucoma, a common optic neuropathy [26,27]. Retinal ganglion cells (RGCs) and their axons form the optic nerve, which relays visual information from the eye to the brain. In glaucoma, these axons deteriorate, leading to visual impairment or even complete loss of vision. Glaucoma is a group of heterogeneous diseases that is frequently associated with elevated intraocular pressure (IOP) or ocular hypertension (OHT), yet glaucoma can manifest at any IOP. Additionally, RGC and optic nerve axon losses may also occur even if the IOP is controlled [28]. Therefore, pursuing neuroprotection in glaucoma independent of modifying IOP, currently a therapeutic lacuna, is a rational strategy [29,30].

The use of retina-targeted E2 delivery via its DHED prodrug is particularly relevant for glaucoma therapy, as over 60% of glaucoma sufferers are females on a global scale [31]. Epidemiological, clinical as well as basic science studies support the notion that estrogen deficiency brought about by menopause or medical conditions is associated with the onset or risk of developing certain types of glaucoma [27,32,33,34,35,36], whereas E2 supplementation protects against RGC loss [15,37], lowers IOP, and modulates aqueous humor outflow [35]. It has also been proposed that an “estrogenic” retina is important for healthy visual functioning [38]. Therefore, when the circulating E2 supply is lacking, targeting E2 to the highly vulnerable hypoestrogenic retina would be of interest translationally due to its promise to ensure therapeutic safety and efficacy.

We have shown previously that DHED eye drops confined the formation of E2 to the retinas of the female and male Brown-Norway (BN) rats as well as New Zealand white rabbits owing to the rapid NADPH-dependent DHED-to-E2 bioconversion at the site of action (Figure 1) [39,40]. To assess neuroprotection while focusing on vision rescue afforded by the DHED-derived E2, we used a male rat model of glaucoma in which hyperosmotic saline injection into an episcleral vein produced OHT [41]. Despite the sustained high IOP, a significantly preserved visual acuity and contrast sensitivity (CS) were achieved in the DHED-treated BN rats. Alongside, the once daily (q.d.) topical DHED treatments also resulted in ameliorating OHT-triggered glaucomatous dysregulations of a multitude of proteins involved in retinal neurodegeneration without increasing circulating E2 levels, even after 12 weeks of q.d. eye drops [42]. We also used retina proteomics relying on mass spectrometry (MS) to characterize the model itself for the first time in terms of identifying retinal protein networks impacted by OHT [43].

In the present study, we utilized another well-established model of RGC injury to evaluate ocular neuroprotection achieved by DHED eye drops in E2-deprived female BN rats. In the optic nerve crash (ONC) paradigm, the inflicted acute injury induces optic nerve axonal trauma, independent of IOP, with characteristic progression of vision loss and ocular neurodegeneration [33,34,35,36,44,45]. Ovariectomy (OVX), on the other hand, has traditionally been used as a model for surgical menopause in women [46]. OVX produces a rapid decline in circulating endogenous E2, because the ovaries are the principal sources of this hormone before menopause [12]. OVX increases the risk of retinal and optic nerve degenerations [34,35,36] by introducing a hypoestrogenic state in the animal. Here, we used a multi-pronged approach including behavioral evaluations of visual function and retinal structure in the ONC model with and without topical DHED treatments, as well as MS-based retina proteomics to survey retinal protein networks impacted by ONC and identify E2-responsive proteins in the context of neurodegeneration and amelioration thereof.

## 2. Results

### 2.1. Assessments of Vison and Retinal Structure in the ONC Model of Surgical Menopause with and Without Topical DHED Treatments

The following experimental groups consisting of middle-aged (9–10 months) BN female rats were used in our studies: Sham animals with a unilateral ONC and OVX animals with ONC followed by treatment with topical DHED (0.1% *w*/*v*) or a vehicle, 20% hydroxypropyl-β-cyclodextrin (HPβCD) in saline [39,40,42]. ONC was performed 8 weeks after Sham or OVX surgery. Immediately after ONC, the animals started receiving q.d. eye drop treatments into the injured eye for 12 weeks. The contralateral eye served as innate control (CL). Behavioral assessments of vision were based on the animal’s optomotor response (OMR) [47] in terms of spatial frequency (SF) and contrast sensitivity (CS). Retinal thickness was measured by spectral-domain optical coherence tomography (SD-OCT).

#### 2.1.1. Vision

**Figure 2:**
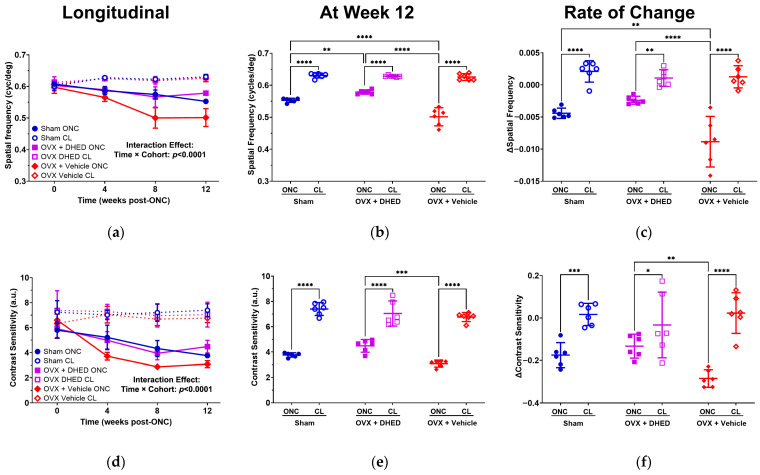
Behavioral assessments of vision based on the OMR of the animal in Sham and OVX BN rats after ONC and neuroprotective intervention. CL eyes were used as innate control. (**a**) SF decreased in ONC eyes relative to CL eyes for all cohorts over 12 weeks; (**b**) SF at 12 weeks after ONC injury as well as DHED or vehicle treatments; (**c**) the rate of change (slope) in SF over the 12-week observational period; (**d**) CS decreased in ONC eyes over 12 weeks for all cohorts; (**e**) CS at 12 weeks; (**f**) the rate of change (slope) was lowest in ONC eyes of OVX + Vehicle animals, and was significantly lower compared to OVX + DHED animals. Similarly to SF outcomes, we did not find a significant difference in CS at 12 weeks, (**e**) or in the rate of change in SF (**f**) between CL of all cohorts (*p* > 0.05 for all comparisons). Results are displayed as mean with error bars representing the 95th confidence intervals. Statistical significance: * *p* < 0.05, ** *p* < 0.01, *** *p* < 0.001, **** *p* < 0.0001 (*n* = 6 per group).

ONC resulted in a significant decline in SF over 12 weeks (RM ANOVA interaction effect, time, and cohort, *p* < 0.0001; Figure 2a). At 12 weeks (Figure 2b), we found that SF was 12.3%, 7.9%, and 19.9% lower in the ONC eye compared to the CL in the Sham (*p* < 0.0001), OVX + DHED (*p* < 0.0001), and OVX + Vehicle (*p* < 0.0001) cohorts. Notably, we found that ONC eyes of OVX + DHED had a significantly higher spatial frequency compared to Sham group (*p* = 0.004) and OVX + Vehicle (*p* < 0.0001), indicating that DHED treatment helped preserve visual function after ONC. To evaluate the impact of DHED treatment on the progressive loss of spatial frequency over 12 weeks, we assessed the slope of spatial frequency over time (Figure 2c). Markedly, we found that loss of spatial frequency (slope) in ONC eyes was the largest in the OVX + Vehicle group compared to Sham (*p* = 0.0004) and OVX + DHED (*p* < 0.0001).

We found a similar trend when examining the CS (Figure 2d–f). There was a significant interaction between time and cohort (*p* < 0.0001) as this readout decreased over the 12-week observational period (Figure 2d). By examining CS 12 weeks after ONC, we found that OVX + DHED had a significantly higher CS in ONC eyes compared to the OVX + Vehicle treatment (*p* = 0.0001; Figure 2e). However, relatively to CL eyes, ONC eyes had a 49.2%, 36.3%, and 54.4% lower CS in the Sham, OVX + DHED, and OVX + Vehicle cohorts (*p* < 0.0001 for all comparisons). We found that the rate of contrast loss (slope) between the CL and ONC eyes was significant in the Sham (*p* = 0.002) and OVX + Vehicle (*p* < 0.0001). In contrast, the rate of CS loss (slope) in OVX + DHED animals was significantly lower (*p* = 0.037) compared to the OVX + Vehicle group of animals.

#### 2.1.2. Retinal Structure

**Figure 3:**
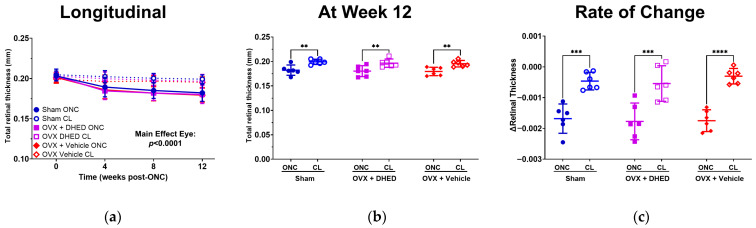
Impact of ONC on retinal thickness in the BN retired breeder female rats after Sham or OVX surgery; (**a**) total retinal thickness decreased in ONC eyes relative the CL eye for all cohorts); (**b**) at 12 weeks, total retinal thickness was significantly lower in ONC compared to CL eyes for all cohorts; (**c**) the rate of retinal thinning (slope) was significantly higher in ONC compared to CL. Results are displayed as mean with error bars representing the 95th confidence intervals. Statistical significance: ** *p <* 0.01, *** *p <* 0.001, **** *p <* 0.0001, *n* = 6 per group.

We found an interaction effect of time and cohort (*p* < 0.0001) as total retinal thickness decreased from baseline over 12 weeks (Figure 3a). At 12 weeks, we found that retinal thickness decreased in ONC eyes by 8.5%, 8.3%, and 8.1% in the Sham (*p* = 0.001), OVX + DHED (*p* = 0.0014), and OVX + Vehicle (*p* = 0.0017) cohorts, respectively (Figure 3b). We did not find a significant difference between the rate of retinal thickness thinning (slope) between the Sham, OVX + DHED, and OVX + Vehicle treatment. However, we did find a significant change in retinal thickness (slope) between ONC and CL for Sham (*p* = 0.034), OVX + DHED *(p* = 0.034), and OVX + Vehicle (*p* = 0.018) over 12 weeks.

### 2.2. Discovery-Driven Retina Proteomics

We employed data-dependent acquisition using nanoflow liquid chromatography coupled to tandem mass spectrometry (nLC–MS/MS) [43] for discovery-driven assessments of the retinal proteome affected by ONC to characterize the model itself as well as to survey the effect of DHED-derived E2 on the ONC eye of E2-deprived animals.

#### 2.2.1. Shotgun Retina Proteomics of the ONC Model of Glaucoma

**Figure 4:**
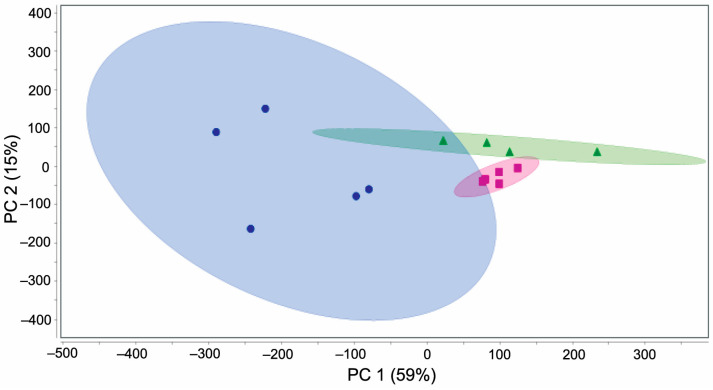
Principal component analysis (PCA) plot showing the similarity and dissimilarity among retinas originating from the three experimental groups (green: Young-Ref; magenta: Sham-CL; blue: Sham-ONC).

ONC and CL retinas from the Sham groups were used in this study. In addition, we included retina tissues from young (2–3-month-old) BN females (Young-Ref) as a reference group, according to our previous study [42]. Our extensive data analysis pipeline involving Proteome Discoverer integrated with the Mascot search algorithm resulted in over 3000 high-confidence protein identifications with at least two identified proteotypic peptides. For a more stringent validation, Scaffold utilizing MSfragger reported nearly 1600 proteins, with an estimated false-discovery rate (FDR) of 0.2% at the protein level and 0.0% at the peptide level using a decoy-based method of FDR estimation (Table S1). Label-free quantitation (LFQ) relied on spectral counting (SC) and Omnibus ANOVA followed by post hoc analysis to obtain proteins with statistically significant differences in their abundance among the three test groups (Table S2a). Surprisingly, protein expressions between Young-Ref and the Sham-CL retinas were insignificant based on ANOVA (Figure 4), with only 23 proteins (approximately 1.6%) being differentially expressed (Table S2b). Therefore, Fisher’s exact test with corrections for multiple tests concerning the Sham-ONC versus Sham-CL retinas only was considered next. We found 318 differentially expressed proteins (DEPs) between the ONC and CL retinas (Table S3). Specifically, 225 proteins were downregulated, and 93 proteins were upregulated as a consequence of ONC using the statistical threshold described in Section 4.9.

To ascertain the overall impact of ONC through system biology, we imported the DEPs to the Ingenuity Pathway Analysis^®^ (IPA^®^) tool. Table 1a summarizes the top molecular and cellular processes. Table 1b lists the top diseases and disorders affected by ONC in the middle-aged, retired breeder BN female retina proteome. Proteins impacted by the induced ONC also triggered more than 150 canonical pathways (Table S4a) and more than 50 significant disease and function networks (Table S4b). The findings were also organized by IPA^®^ into protein interaction networks focusing on ONC-induced retinal damage (Table S4c).

**Figure 5:**
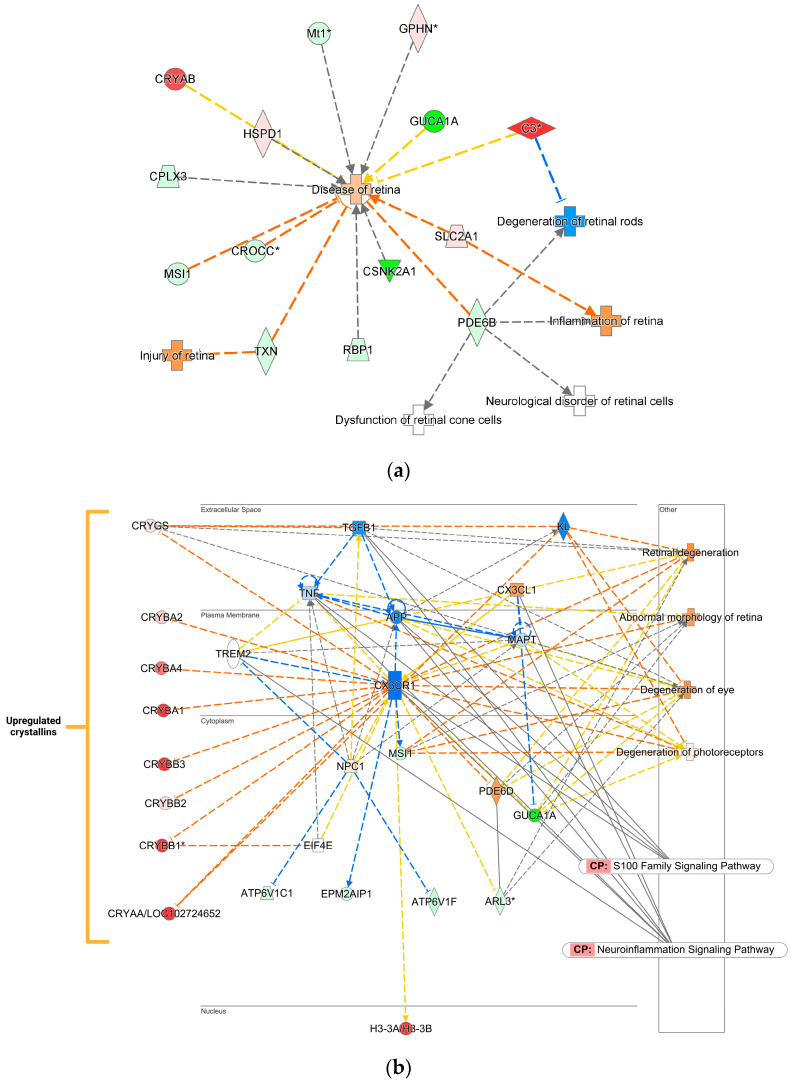
IPA^®^ mapping of ONC-impacted proteins in the Sham BN rat retina: (**a**) A subset of these proteins was linked to a disease and function network associated by the knowledge base on retinal disease, inflammation of the retina, degeneration of retinal rod and cone cells, and neurological disorder or retinal cells. Blue dashed line: inhibition/decrease; orange dashed line: activation/increase; yellow dashed line: cannot be predicted. (**b**) An IPA^®^ protein interaction network linked to cellular movement, hematological system development and function, and immune cell trafficking also shows crystallins’ regulation and association with retinal disease processes. CP—canonical pathway; red—upregulation; green—downregulation; shade of color indicates the extent of change in expression. Solid gray line—direct relationship; dashed gray line—indirect relationship; blue dashed line—inhibition/decrease; orange dashed line—activation/increase; yellow dashed line—cannot be predicted; blue solid line—inhibition. Asterisks indicate that multiple protein identifiers (isoforms) in the input file were mapped to the same gene by IPA^®^. Other (placed in the rectangular box on the right): functions and diseases associated with the indicated elements of the network. Abbreviations of proteins are listed in Table S7a,b.

In Figure 5a, we highlight several of the prominent diseases and functions associated pathways that were affected after ONC, including disease of the retina, degeneration of retinal rod and cone cells, inflammation and injury of the retina, and neurological disorder of retinal cells. Based on this regulation pattern, IPA^®^’s molecular predictor activity (MAP) tool predicted the elevation of retinal disease in the ONC eye. Figure 5b, on the other hand, represents a top protein interaction network constructed by IPA^®^. This network is related to cellular movement, hematological system development and function, and immune cell trafficking. This network also shows the regulation of various crystallins, particularly the upregulation of multiple crystallins in response to ONC-induced damage. Figure 5b also indicates the stimulation of the S100 family signaling and neuro-inflammation signaling pathways. These signaling cascades orchestrate various stress-induced reactions in the cell. This network shows the activation of different disease and function pathways as predicted by the MAP tool in IPA^®^, such as eye and retinal degenerations and photoreceptor degeneration.

#### 2.2.2. Discovery-Driven Retina Proteomics in the Optic Nerve Crush Model of Glaucoma After Tropical DHED Treatments

**Figure 6:**
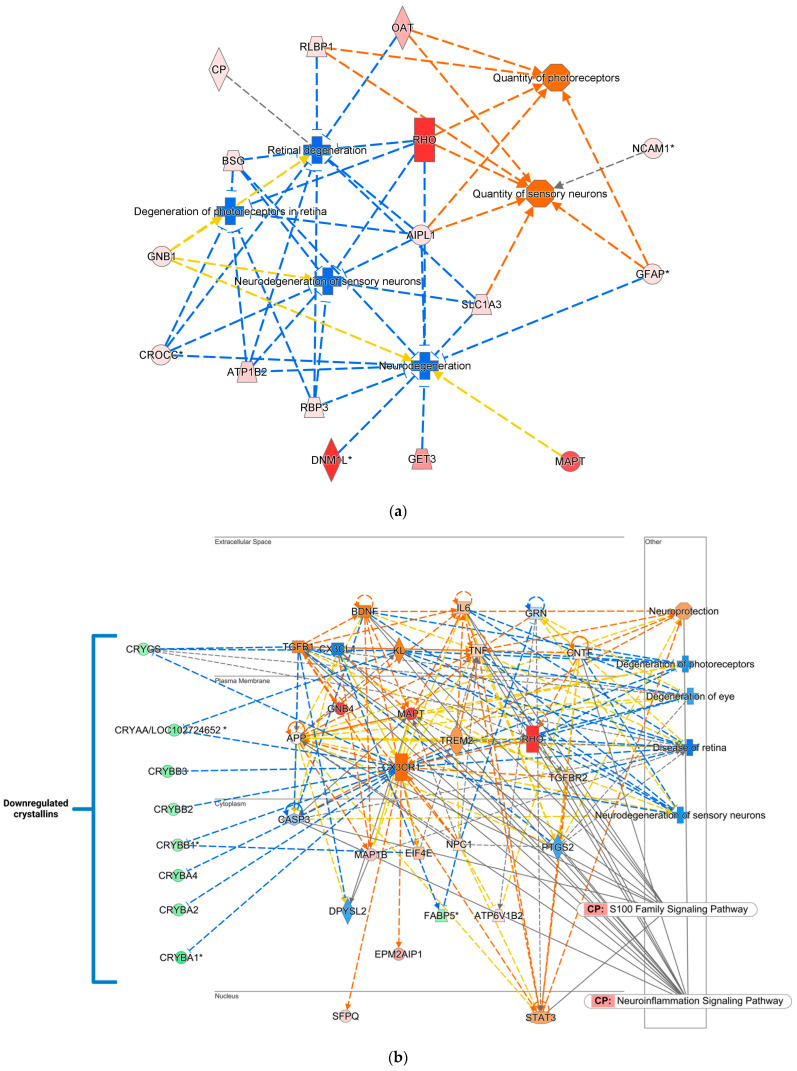

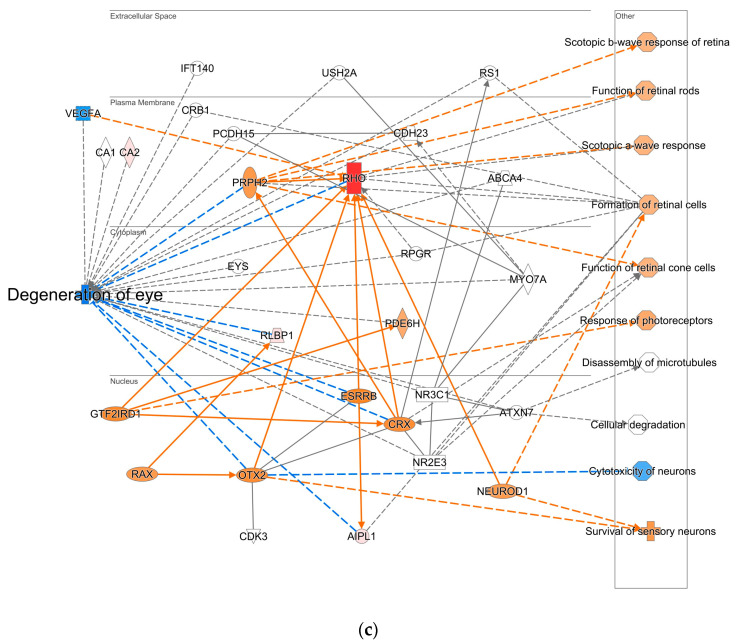
IPA^®^-based illustration of the neuroprotective effects of retina-targeted E2 treatment via its DHED prodrug in the OVX + DHED group: (**a**) A subgroup of these proteins was mapped to a physiological function and disease network pinpointing the associated proteins with their regulation pattern by the knowledge base assigning functions related to neurodegeneration, the number of photoreceptors and sensory neurons, degeneration of photoreceptors, neurosensory neurons, and the retina. Blue dashed line: inhibition/decrease; orange dashed line: activation/increase; yellow dashed line: cannot be predicted. (**b**) An IPA^®^ protein interaction network predicted inhibition of eye degeneration mediated by our therapeutic intervention. This IPA^®^-mapped protein interaction network also represents cell death and survival, cellular compromise, and neurological disease, and shows the regulation of different forms of neuroprotective crystallins. (**c**) An IPA^®^ ML protein interaction network predicted inhibition of eye degeneration mediated by DHED-derived E2 treatment. CP—canonical pathway; red—upregulation; green—downregulation; shade of color indicates the extent of change in expression. Solid gray line—direct relationship; dashed gray line—indirect relationship; blue dashed line—inhibition/decrease; orange dashed line—activation/increase; yellow dashed line—cannot be predicted; orange solid line—direct activation/increase. Asterisks denote that multiple protein identifiers (isoforms) in the input file were mapped to the same gene. Other (placed in the rectangular box on the right): functions and diseases associated with the indicated elements of the network. Abbreviations of proteins are listed in Table S7c–e.

Next, we compared the ONC retinas from the OVX + DHED and OVX + Vehicle groups. Proteomics-based analyses revealed that 295 proteins were significantly different (43 downregulated and 252 upregulated) between the two study groups (Table S5). Some prominent observations of the shotgun proteomics are listed in Table 2 and illustrated in Figure 6. Table 2a represents the most significant molecular functions, such as cellular morphology-, assembly-, development-, growth-, and proliferation-related functions, to be regulated by the neuroprotective topical treatments, whereas Table 2b lists the top physiological functions affected by the retina-targeted E2 therapy. These all relate to nervous system, organismal, tissue, and embryonic development and function.

IPA^®^ returned more than 150 significantly affected canonical pathways (Table S6a), over 50 significant disease and function networks (Table S6b), and 17 interaction networks (Table S6c). Figure 6a shows several physiological functions and disease features impacted by E2 treatments via the DHED eye drops to counteract ONC-induced retinal damage in the OVX animals. These included a decrease in neurodegeneration, retinal and photoreceptor degenerations, and an increased quantity of photoreceptors and sensory neurons. Based on this regulation pattern by the MAP tool, IPA^®^ predicted amelioration of the retinal disease in the ONC model via our retina targeted E2 therapy. Figure 6b signifies a crystalline-dominated network representing embryonic development, nervous system development and function, and organ development according to IPA^®^’s knowledge base. The MAP tool of IPA^®^ projected inhibition of damage to the nervous system, photoreceptors, and sensory neurons linked to the downregulated pattern of the different crystallin isoforms. The top canonical signaling pathways triggered by this interaction network are neuroinflammation and S100 family signaling pathways, which are involved in various neurodegeneration and neuroprotection aspects (Table S6a). Figure 6c represents a disease pathway created through machine learning (ML) by mining the QIAGEN knowledge base. These pathways highlight crucial proteins that affect a single disease and its associated phenotypes, thus retinal degeneration in the present context. By using a combination of ML and other heuristic approaches, our approach allowed us to prioritize critical proteins that affect retinal degeneration and create a link between the disease and potential phenotypes. Overall, Figure 6 shows the various aspects of neuroprotection exerted by E2 upon topical application of its DHED prodrug following RGC injury.

## 3. Discussion

ONC is a model for optic neuropathies that induce loss of RGCs independent of IOP [48]. In this model, the projecting RGC axons are crushed posterior to the eye to induce a mild injury within the optic nerve, causing RGC death, inflammation, and vision loss [34,36,49]. Another benefit of this mild ONC model is that it induces gradual and progressive loss of visual function caused by the chronic neurodegeneration of RGCs. Our study utilized a mild ONC to mirror the gradual, progressive vision loss due to acute ON injury. Studies suggest that E2 deprivation may promote vulnerability to ocular diseases, with earlier onset of menopause positively correlated with earlier onset of glaucoma [32,33,34,35,36]. Therefore, estrogen-regulated signaling pathways may play a role in protecting the eye from optic neuropathies; however, direct use of E2 as a neurotherapeutic is hindered due to the numerous off-target side effects in the periphery [13,22,23,24]. To combat these limitations, we used DHED, a unique bioprecursor prodrug of E2 developed in our laboratory [25,39,40]. DHED is only covered into E2 within the central nervous system, making the retina a site-specific target of E2 and ensuring therapeutic safety. Previously, we have shown the benefits of DHED eye drops for the protection of the RGCs against OHT-induced degradation in male BN rats [42]. In this study, we assessed the utility of DHED eye drops in the mild ONC model of RGC injury following OVX, thus in E2-deprived BN female rats. Our novel study focused on the preservation of vision function, which we complemented with MS-based discovery-driven retinal proteomics.

Our previous work has shown that OHT or ONC following OVX leads to worse visual function compared to Sham-operated female rats [33,35,36]. We expected that the additional decline in visual function in these injury models was related to the loss of circulating E2 following OVX. Similarly to our previous work [36], we found that OVX animals receiving vehicle treatment had the lowest SF after ONC (Figure 2a–c). However, OVX animals receiving DHED had significantly preserved SF and CS relative to OVX animals receiving the vehicle treatment only (Figure 2). Further, we found that the rate of loss of SF and CS was slower in OVX animals receiving DHED compared to OVX animals receiving vehicle treatment. This highlights that topical DHED treatments slowed the progression of visual function loss after RGC injury and highlights the potential benefits of DHED treatment for preserving vision in glaucoma. Next, we found that ONC caused thinning of the retina after ONC injury compared to the CL eyes (Figure 3). However, we did not find significant differences between the Sham and OVX cohorts. These findings are consistent with our previous work [36] suggesting that OCT measurements at 4-week intervals may not be sensitive enough to detect the impact of treatment or that these changes are too subtle to detect with OCT. It is possible that examining retinal thickness at earlier time points (1 or 2 weeks) after injury will improve our ability to detect differences due treatment or menopausal status.

Our shotgun retina proteomics studies were congruent with our behavioral assessments of vision and retinal thickness measurements. Induction of a unilateral ONC in the Sham rats led to the gradual loss of visual performance by week 12 (Figure 2) in comparison with the CL eye. However, employing a contralateral eye to serve as the control has been suggested to not be a “true” control [50]. We addressed this caveat by including the young reference animal in the analysis, as they are independent of any retinal damage and potential age-related factors. The gross comparison showed extensive similarities between the young (2–3-month-old) reference animals and the CL eye of the middle-aged rats (Figure 4), with only about a 1.6% difference in the gross number of dysregulated proteins (Table S2b). Furthermore, our PCA indicated that the Young-Ref and the Sham-CL retinas were strikingly similar, differing all the while from the ONC retinas (Figure 4).

In agreement with the reduced SF and CS (Figure 2 and Figure 3), the proteomic analysis revealed significant differences between the ONC and CL eyes, with many DEPs associated with neurodegeneration, inflammation, and metabolic disease (Table S4b). Furthermore, some DEPs are related to functions associated with specific cell types, such as neurons and photoreceptors. Based on the submitted DEPs, IPA^®^ predicts the promotion of disease and neurodegeneration of the retina after ONC, suggesting that RGCs’ death continues even after 12 weeks post-injury (Figure 5). However, these predicted processes in the ONC vs. uncrushed CL eye were the opposite in the DHED-treated animals, where degeneration was predicted to be inhibited (Figure 6), reflecting the benefit on visual function over our experimental period (Figure 2).

Most strikingly, various crystallin (CRY) proteins from the alpha, beta, and gamma families were significantly upregulated in the ONC retina (Figure 5). Subtypes of the CRY proteins from all three families have been shown to possess neuroprotective effects against glaucoma [51]. CRY-α are heat-shock proteins most associated with maintaining and protecting the ocular lens from oxidative stress [52]. However, reports of anti-apoptotic activity of CRY-α through inhibition of mitochondrial pore-complex formation and modulation of pro-growth signaling pathways suggest additional protective effects [53]. Similarly, CRY-β and CRY-λ have shown protective effects in retinal neurons in response to disease with an injection of CRY-βB2 and CRY-λB protecting RGCs in uveitis and glaucoma models [51,54].

The elevated expression of the CRY proteins may be a compensatory mechanism due to the ONC. Other disease models have shown elevation of CRY proteins in glaucomatous retina and specifically in photoreceptors exposed to UV and drusen [55,56,57]. Furthermore, CRY-α and CRY-β protein expressions are elevated in RGCs after axonal injury [58,59]. Conversely, topical treatment with DHED reduced the expression of all crystallin proteins compared to the vehicle control (Figure 6b). The suppression may be related to the DHED-derived E2 providing resistance to cellular stress associated with tissue damage through other mechanisms. Furthermore, the expression of crystallins can differ depending on the proximity to the initial injury. In another study utilizing the ONC model, the expression of several crystallins was upregulated initially; however, by week 12, it was normalized back to base levels before IOP elevation [51].

Several proteins, while not found in our initial list of DEPs, were predicted by IPA^®^ to be dysregulated. These may serve as future targets of interest. For example, the neuroprotective “anti-aging” protein klotho (KL) was included as part of the pathway related to the degeneration of the eye (Figure 5b and Figure 6b). Studies have shown that KL possess neuroprotective effects in the context of neurodegenerative stress induced by axotomy with reduced expression found in patients prone to pseudoexfoliation-induced glaucoma [60,61,62]. KL was predicted to be suppressed in the ONC retina, potentially indicative of continued neuronal stress (Figure 5b). The opposite was predicted by IPA^®^ in the DHED-treated animals, as KL expression was predicted to be activated, potentially combating the stress induced by ONC (Figure 6b). Whether E2 directly or indirectly upregulates KL is unknown but warrants further investigation given its role in neuroprotection.

Additionally, IPA^®^ predicted elevated activation of brain-derived neurotrophic factor (BDNF, Figure 6b), widely considered a neuroprotective protein and the focus of gene therapy for glaucoma [63]. RGCs specifically express the tropomyosin receptor kinase B, through which BDNF contributes to neuroprotection and cell survival [64]. While BDNF was not among the list of DEPs, E2 is linked to BDNF expression and its signaling cascade (Figure 6b) [65]. Altogether, the upregulation of KL and BDNF may indicate pro-survival and growth of the retinal neurons in response to the DHED-derived E2.

Immune-associated DEPs in the ONC versus uncrushed retinas were related to both innate and active immunity including neutrophil degranulation, MHC Class 2 antigen presentation, IL-1 signaling, and CTLA4 signaling in cytotoxic T lymphocytes. These were all dysregulated by the ONC (Table S4b). However, many of these pathways were predicted to be suppressed according to IPA^®^ in the ONC retina. While ONC may induce rampant localized inflammation and immune cell recruitment initially, 12 weeks after the acute insult may give enough time for the retinal axons to transition towards anti-inflammation and wound healing; therefore, the captured protein expression may show that some aspects of the immune system have transitioned towards dampening the immune response.

On the other hand, protein complement C3 (which is generally considered pro-inflammatory) was significantly upregulated in the Sham-ONC retina, as shown in Figure 5a. Classically, C3 is a mediator of the complement system in response to a bacterial pathogen when cleaved by C3 convertase into its subunits, which in turn can signal apoptosis, inflammation, and active immune response [66]. Several studies suggest that elevated expression of C3 is associated with RGC neurotoxicity depending on the type of ocular neurodegenerative model used. In another study utilizing the ONC rat model, C3 mRNA expression was upregulated in the injured optic nerve and remained elevated for 28 days. The uncrushed contralateral optic nerve did not express elevated mRNA of the C3 [67]. However, that study did not include protein validation or other molecular analysis of C3. Likewise, a study using the experimental autoimmune encephalomyelitis (EAE) model found elevated C3 was secreted by neurodegenerative astrocytes [68,69]. Finally, transgenic C3 knockout mice had more RGCs after retinal ischemia–reperfusion than the wildtypes, citing potential neurodegenerative effects towards RGCs [70]. However, a study using an ocular hypertensive model for glaucoma found the opposite effect, where C3 was protective in that model; instead, protein complement C1q was considered by the authors to be neurodegenerative to RGCs [71]. Therefore, C3 may serve as a positive marker for damage associated with the optic nerve, as we found C3 remained elevated in the ONC retina 12 weeks after ONC.

Treatment with DHED downregulated neuroinflammatory signaling, necroptosis, and immunogenic cell death signaling. This suggests that E2 bolsters anti-inflammatory processes and may promote wound healing in the optic nerve. According to the IPA^®^, protein–interaction network, upregulation of anti-inflammatory proteins, such as triggering receptor expressed on myeloid cells 2 (TREM2), dihydropyrimidinase-related protein 3 (DPYSL3), and signal transducer and activator of transcription 3 (STAT3), was regulated by E2 [72,73,74] (Figure 5a). This supports the general neuroprotection regulated by E2, as neuroinflammation by dysregulated glial cells may contribute to RGC death [75].

Several photoreceptor-associated proteins were downregulated in the ONC retina; these include guanylate cyclase activator 1A (GUCA1A1), retinol binding protein 1 (RBP1), complexin-3 (CPLX3), and phosphodiesterase 6B (PDE6B) (Figure 5) [76,77,78,79]. Downregulation of these photoreceptor proteins may suggest dysfunction in the photoreceptors; however, many of these proteins have yet to be investigated in ocular neurodegenerative diseases. We found that proteins for photoreceptor function—such as rhodopsin (RHO), interphotoreceptor matrix proteoglycan 2 (IMPG2), guanine nucleotide-binding proteins 1 (GNB1), and retinol-binding protein 3 (RBP3)—were upregulated by the DHED-derived E2 [80,81,82] (Figure 6), suggesting improved function and survival of the photoreceptors following ONC. Interestingly, RBP3 expression was inversely correlated with diabetic retinopathy, suggesting a protective effect against the disease potentially through interaction with vascular endothelial growth factor A (VEGF) [83]. RBP3 may serve as a potential biomarker for neuroprotection in the ONC model. Moreover, inhibition of VEGF was predicted to be protective against ONC, and overexpression can potentially lead to pathological angiogenesis [84,85]. While E2 directly regulates VEGF transcription and is a potential concern for estrogen-based ocular therapies [86], the ability of targeted delivery of E2 to prevent off-target tissues from expressing VEGF is unknown. Interestingly, there is predicted suppression of VEGF by IPA in our networks (Figure 6c).

Additionally, several DEPs have little or no information regarding their role in neurodegeneration. Gephyrin (GPHN), a protein complex that anchors receptors, such as glycinergic and GABAergic, in retinal neurons, was found to be upregulated in the ONC retina [87] (Figure 6a). Only one study reported GPHN expression changing in the context of an autoimmune model of glaucoma, which is the opposite of what we found in our study [88]. While DPYSL3 is involved in glaucoma, dihydropyrimidinase-related protein 5 (DPYSL5) has little information regarding its role in ocular diseases. The role of DPYSL5 is implicated in axonal guidance, and its mRNA expression is downregulated in early glaucoma. In our list of DEPs, this protein was upregulated in the DHED-treated animals, potentially serving as a biomarker for modulating neurodegeneration after ONC [89] (Table S5). Similarly, ciliary rootlet coiled-coil rootletin (CROCC) was upregulated in the DHED-treated animals but downregulated in the ONC versus CL dataset (Tables S3 and S5). The role of CROCC in glaucoma remains to be studied; however, it may serve as a biomarker for E2 response or other neuroprotection interventions in the retina.

## 4. Materials and Methods

### 4.1. Chemicals and Reagents

DHED was prepared in our laboratory through the stereospecific oxidation of E2, as reported before [90]. Chemicals for the synthesis were purchased from Millipore Sigma (St. Louis, MO, USA). Sequencing-grade trypsin was ordered from Promega (Madison, WI, USA). Optima^®^ LC/MS-grade chromatographic solvents and Neomycin cream (Certi-Sporyn) were supplied by Thermo Fisher Scientific (Waltham, MA, USA). Ketamine, xylazine, 1% carboxymethylcellulose sodium (Refresh Celluvisc), Atipamezole (Antised), 1% tetracaine hydrochloride eye drop solution (Minims), and tropicamide (1%) eye drops were all procured from the Atlanta VA Pharmacy (Atlanta, GA, USA).

### 4.2. Animals

All procedures conformed to the ARVO Statement for the Use of Animals in Ophthalmic and Vision Research and were approved by the Institutional Animal Care and Use Committee of the Atlanta VA Healthcare System (approval number: #IACUC-V011-20 approved on 28 August 2020) and the University of North Texas Health Science Center (approval number: #IACUC-2023-0012 approved on 2 May 2023). Middle-aged Sham and OVX (*n* = 18; 9–10 months old, weighing 220–260 g) as well as intact young (2–3 months old, weighing 200–210 g) BN female rats were purchased from Charles Rivers Laboratories (Wilmington, DE, USA). All Sham and OVX surgeries were performed by the supplier. Animals were kept in standard housing with ad libitum access to food and water.

### 4.3. Induction of ONC

Unilateral ONC was performed on the anesthetized animal (ketamine and xylazine at 60 and 7.5 mg/kg body weight, respectively, i.p.) eight weeks after Sham or OVX surgery, as described before [33,35,91]. Briefly, the animal was moved onto a heating pad and both eyes received topical anesthetic (1% tetracaine) and the right eye was visualized under a surgical microscope. The optic nerve was accessed by dissection along the central nasal region of the eye through the conjunctiva and then moving posteriorly past the ocular muscle and orbital fat. Then, the optic nerve was isolated and closed by self-closing forceps around the nerve for 10 s to induce a mild ONC. After surgery, the eye was treated with a topical neomycin. The contralateral eye (CL) served as an innate reference and was kept moist using 1% carboxymethylcellulose sodium throughout the procedure. All animals received atipamezole (2.1 mg/kg body weight) to reverse the effects of the anesthesia.

### 4.4. Topical DHED Treatment

Sterilized DHED (0.1% *w*/*v*) eye drops were prepared as reported before [39,40] in 20% *w*/*w* HPβCD in saline vehicle. OVX animals that underwent ONC (*n* = 6, each) either received DHED or vehicle eye drops (10 μL) into the ONC eye for 5 days per week from Monday to Friday, between 9 and 11 a.m., for 12 weeks starting immediately after ON injury. Within 24 h after the last treatment, the animals were euthanized, and tissues were collected and stored at −80 °C until further processing.

### 4.5. Behavioral Assesments of Visual Function

The visual function based on SF and CS was assessed utilizing the OptoMotry system introduced 20 years ago by Cerebral-Mechanics (Cerebral-Mechanics, Lethbridge, AB, Canada) according to previous reports [33,35,37,92]. In brief, the animals were placed on an elevated platform in the center of a virtual reality chamber consisting of four monitors that display vertical sinewave gratings that rotate at a constant speed of 12 o/s. A masked observer monitored the animal through a video camera mounted above the platform to track the rat’s clockwise or counterclockwise head turn. The animal’s SF threshold was calculated by setting the vertical bands to a 100% contrast and adjusting the spatial frequency between 0 and 0.68 c/D (cycle per degree) until the rat no longer responded. To assess CS, the SF was set to 0.064 c/D while the contrast was decreased from 100% until the animal no longer displayed a positive response. CS is reported as the reciprocal of the Michelson contrast as previously described [33,35]. Baseline SF and CS values were collected before inducing ONC, and then again at 4, 8, and 12 weeks post-ONC. In this behavioral test of vision, each eye is assessed independently [92].

### 4.6. In Vivo Assesment of Retinal Structure

Total retinal thickness was evaluated with a Bioptigen Envisu R4300 SD-OCT system from Leica Microsystems (Buffalo Grove, IL, USA). Readouts were taken at baseline then at 4, 8, and 12 weeks post-ONC, as reported before [33,35]. Briefly, both eyes of the anesthetized rat (ketamine and xylazine at 60 and 7.5 mg/kg body weight, respectively, i.p.) received topical anesthesia (tetracaine, 1%) and the pupils were dilated with tropicamide (1%). Then, four 3 mm radial scans (1000 A-scans per B-scan and 10 frames per B-scan) centered at the optic nerve head in both ONC and CL eyes were acquired [33,35]. Thickness measurements within 0.3 mm of the ON head were excluded. Total retinal thickness was measured from the inner limiting membrane to the retinal pigment epithelium. The average of all the thickness values from each radial scan was taken and reported as a single retinal thickness for each eye.

### 4.7. Statistical Analyses

All data were determined to be normally distributed before performing the appropriate statistical test. For SF, CS, and retinal thickness outcomes, we used a 3-way repeated measures ANOVA design to determine differences between our cohorts. The repeated measures were eye (CL vs. ONC) and time (baseline, 4-week, 8-week, or 12-week). To examine differences at 12 weeks, we used a 2-way repeated measures ANOVA design with a Sidak post hoc analysis to determine significant differences between paired eyes (CL vs. ONC) and cohorts (GraphPad Prism Version 8.0, GraphPad Software, San Diego, CA, USA, www.graphpad.com). We also compared the rate of change in SF and CS after ONC in each cohort using a linear regression. In brief, we calculated the slope of the change in these readouts per week. We then determined the differences in the slope of CL and ONC eyes between cohorts (Sham, OVX + DHED, and OVX + Vehicle) using a one-way ANOVA with a Sidak post hoc. For example, a negative slope indicates that the measurement (i.e., SF or CS) decreased after ONC, and a larger slope value indicates a quicker rate of change in that measurement every 4 weeks.

### 4.8. Shotgun Proteomics

Protein extraction was based on customary processing for trypsin digestion, followed by sample cleanup with C18 cartridges, as stated before [40,42,93]. Samples containing 1 µg/µL of protein were reconstituted in 5% aqueous acetonitrile containing 0.1% HCOOH. Data-dependent nanoflow liquid chromatography coupled to electrospray tandem MS (nLC-ESI-MS/MS) was run on an LTQ Orbitrap Velos Pro mass spectrometer connected to EASY-nLC 1000 systems (both from Thermo Fisher Scientific, San Jose, CA, USA). We used a Phenomenex bioZen 2.6 μm Peptide XB-C18 nano column (15 cm × 75 μm i.d., Phenomenex, Torrance, CA, USA) for nLC, with connection to the EASY-Spray source of the mass spectrometer provided by a 7 μm i.d. nanoflow EASY-Spray emitter (both from Thermo Fisher Scientific, San Jose, CA, USA). The source voltage was 2.2 kV, and the ion-transfer tube temperature was set to 275 °C. Two solvents were used to create a 100 min binary solvent gradient: solvents A and B were water and acetonitrile, respectively, containing 0.1% HCOOH. An amount of 3 µL of the sample solution was injected. At the beginning of each run, column equilibration was performed by maintaining constant column pressure at 450 bar for 22 min with 100% A. Peptides were eluted at a 300 nL/min flow rate with the following solvent gradient program: (i) 5 min isocratic at 3% B; (ii) linear program to 40% B over 75 min; (iii) isocratic at 40% B for 5 min; (iv) then to 85% B over 2 min; (v) isocratic at 85% B for 3 min; and (vi) resetting to 3% B in 10 min. During elution, full-scan mass spectra (MS) were acquired with a nominal resolution of 60,000 (at *m*/*z* 400) in the Orbitrap, and up to 20 MS-dependent MS/MS spectra were obtained in the ion trap. Each full MS/MS spectrum was acquired using collision-induced dissociation set at 35 of only multiply charged ions (*z* ≥ 2). After selecting the ion to be fragmented, dynamic exclusion was set for 60 s.

### 4.9. MS/MS Data Analysis

MS/MS spectra were then searched against the UniProt protein sequence database (species: Rattus norvegicus, 2022; 36,254 entries) using both MSFragger (The Nesvizhskii Lab, 1301 Catherine, 4237 Medical Science I, Ann Arbor, MI 48109, USA) and Mascot search algorithm (version 2.6.2, Matrix Science, Boston, MA, USA) within the Proteome Discoverer (version 2.4, Thermo Fisher Scientific, San Jose, CA, USA) software. Parent ion tolerance of 25 ppm, fragment ion mass tolerance of 0.80 Da, and one missed cleavage were set as search filters. Fixed modifications included carbamidomethylation of C, with variable modifications for oxidation of M, as well as deamidation of N and Q. Search results were validated to meet decisive criteria of protein identifications using the Peptide and Protein Prophet algorithms [94] requiring >95% at peptide level and >99% at protein level probabilities, and at least two identified unique peptides for each protein using the Scaffold software (version 5.3.3; Proteome Software Inc., Portland, OR, USA).

LFQ relied on SC [40,43,93] built into the Scaffold 5 software, and *p* < 0.05 was considered significantly different using Fisher’s exact tests [95] upon comparing protein abundances for all pairwise comparisons between the Sham-ONC and Sham-CL retinas and OVX + DHED + ONC vs. OVX + Vehicle ONC. Benjamini–Hochberg (BH) corrections were applied for multiple testing and a ≥1.5-fold difference in SCs for all the above-mentioned groups was set as the threshold of significant change in protein abundances. Missing values, if any, were handled using Scaffold’s default method and settings. PCA plots were generated by loading the peptide and protein results from Scaffold 5 to Scaffold Quant (version 5.0.3; Proteome Software Inc., Portland, OR, USA).

### 4.10. Bioinformatics

The identified ONC-regulated proteins, as well as DHED-derived E2-responsive proteins, were submitted to IPA^®^ (QIAGEN, Redwood City, CA, USA) to derive bioinformatics annotations along with potential protein interaction networks, as well as associated biological functions and processes. Overlaps of *p*-values were reported from IPA^®^’s calculations using the right-tailed Fisher exact test [95]. Z-scores were generated for regulated functions, and employing the MAP tool integrated into the software allowed us to assume the directionality of the triggered signaling cascades’ molecular, physiological, and disease-related functions.

## 5. Conclusions

We found that visual impairments and altered proteomics after surgical menopause, via OVX followed by ONC, can be ameliorated by retina-targeted delivery of E2 via its DHED prodrug. This suggests that supplying E2 to the retina after menopause may be a novel treatment for preserving visual function, and DHED may offer a unique approach to reducing the off-target impact and concerns regarding the chronic use of E2.

## Figures and Tables

**Table 1:** (**a**) Molecular and cellular processes as well as (**b**) diseases and physiological functions represented by the significantly regulated retina proteins in the Sham-ONC group compared to Sham-CL.

(a)		
Represented Processes	*p*-Value of Overlap	Number of Associated Molecules
Cellular Assembly and Organization	3.69 × 10^−2^–4.58 × 10^−4^	12
Cellular Movement	2.47 × 10^−2^–9.43 × 10^−4^	4
DNA Replication, Recombination, and Repair	1.24 × 10^−2^–2.8 × 10^−3^	3
Cell-To-Cell Signaling and Interaction	3.69 × 10^−2^–3.1 × 10^−3^	15
Cellular Function and Maintenance	3.69 × 10^−2^–4.1 × 10^−3^	8
**(b)**		
**Associated Diseases**	***p*-Value of Overlap**	**Number of Associated Molecules**
Neurological Disease	4.88 × 10^−2^–1.96 × 10^−6^	53
Organismal Injury and Abnormalities	4.88 × 10^−2^–1.96 × 10^−6^	59
Skeletal and Muscular Disorders	2.42 × 10^−2^–1.26 × 10^−5^	24
Psychological Disorders	4.88 × 10^−2^–2.39 × 10^−5^	37
Hereditary Disorder	2.47 × 10^−2^–5.50 × 10^−5^	21

**Table 2:** (**a**) Molecular and cellular processes, as well as (**b**) physiological functions represented by the DEPs in the ONC retinas from the OVX + DHED and OVX + Vehicle groups.

(a)		
Represented Process	*p*-Value of Overlap	Number of Associated Molecules
Cell Morphology	4.98 × 10^−2^–3.54 × 10^−7^	39
Cellular Assembly and Organization	4.98 × 10^−2^–3.54 × 10^−7^	34
Cellular Development	4.98 × 10^−2^–3.54 × 10^−7^	28
Cellular Function and Maintenance	4.22 × 10^−2^–3.54 × 10^−7^	32
Cellular Growth and Proliferation	4.98 × 10^−2^–3.54 × 10^−7^	27
**(b)**		
**Associated Function**	***p*-Value of Overlap**	**Number of Associated Molecules**
Nervous System Development and Function	4.98 × 10^−2^–3.54 × 10^−7^	63
Organismal Development	4.98 × 10^−2^–3.54 × 10^−7^	49
Tissue Development	4.98 × 10^−2^–3.54 × 10^−7^	42
Tissue Morphology	4.98 × 10^−2^–4.52 × 10^−7^	39
Embryonic Development	4.98 × 10^−2^–7.83 × 10^−6^	39

## Data Availability

The mass spectrometry proteomics data have been deposited to the ProteomeXchange Consortium via the PRIDE [96] partner repository with dataset identifiers PXD059520 and 10.6019/PXD059520.

## Supplementary Materials

The following supporting information can be downloaded at https://www.mdpi.com/article/10.3390/ijms26051846/s1.
